# Identifying responders to vagus nerve stimulation based on microstructural features of thalamocortical tracts in drug-resistant epilepsy

**DOI:** 10.1016/j.neurot.2024.e00422

**Published:** 2024-07-04

**Authors:** Alexandre Berger, Michele Cerra, Vincent Joris, Venethia Danthine, Benoit Macq, Laurence Dricot, Gilles Vandewalle, Nicolas Delinte, Riëm El Tahry

**Affiliations:** a Epilepsy and Neurostimulation Lab, Institute of Neuroscience (IoNS), Department of Clinical Neuroscience, Catholic University of Louvain, 1200, Brussels, Belgium; b Synergia Medical SA, 1435, Mont-Saint-Guibert, Belgium; c Sleep and Chronobiology Lab, GIGA-Cyclotron Research Center-In Vivo Imaging, University of Liège, 4000, Liège, Belgium; d Institute of Information and Communication Technologies, Electronics and Applied Mathematics (ICTEAM), Catholic University of Louvain, 1348, Louvain-la-Neuve, Belgium; e Politecnico di Torino, Department of Control and Computer Engineering, Corso Duca Degli Abruzzi 24, 10129, Torino, Italy; f Cliniques Universitaires Saint-Luc (CUSL), Department of Neurosurgery, 1200, Brussels, Belgium; g Center for Refractory Epilepsy, Cliniques Universitaires Saint-Luc (CUSL), Department of Neurology, 1200, Brussels, Belgium

**Keywords:** Thalamocortical tracts, Vagus nerve stimulation, Epilepsy, Magnetic resonance imaging, Biomarker, Support vector machine

## Abstract

The mechanisms of action of Vagus Nerve Stimulation (VNS) and the biological prerequisites to respond to the treatment are currently under investigation. It is hypothesized that thalamocortical tracts play a central role in the antiseizure effects of VNS by disrupting the genesis of pathological activity in the brain. This pilot study explored whether *in vivo* microstructural features of thalamocortical tracts may differentiate Drug-Resistant Epilepsy (DRE) patients responding and not responding to VNS treatment. Eighteen patients with DRE (37.11 ​± ​10.13 years, 10 females), including 11 responders or partial responders and 7 non-responders to VNS, were recruited for this high-gradient multi-shell diffusion Magnetic Resonance Imaging (MRI) study. Using Diffusion Tensor Imaging (DTI) and multi-compartment models - Neurite Orientation Dispersion and Density Imaging (NODDI) and Microstructure Fingerprinting (MF), we extracted microstructural features in 12 subsegments of thalamocortical tracts. These characteristics were compared between responders/partial responders and non-responders. Subsequently, a Support Vector Machine (SVM) classifier was built, incorporating microstructural features and 12 clinical covariates (including age, sex, duration of VNS therapy, number of antiseizure medications, benzodiazepine intake, epilepsy duration, epilepsy onset age, epilepsy type - focal or generalized, presence of an epileptic syndrome - no syndrome or Lennox-Gastaut syndrome, etiology of epilepsy - structural, genetic, viral, or unknown, history of brain surgery, and presence of a brain lesion detected on structural MRI images). Multiple diffusion metrics consistently demonstrated significantly higher white matter fiber integrity in patients with a better response to VNS (p_FDR_ < 0.05) in different subsegments of thalamocortical tracts. The SVM model achieved a classification accuracy of 94.12%. The inclusion of clinical covariates did not improve the classification performance. The results suggest that the structural integrity of thalamocortical tracts may be linked to therapeutic effectiveness of VNS. This study reveals the great potential of diffusion MRI in improving our understanding of the biological factors associated with the response to VNS therapy.

## Introduction

Epilepsy is characterized by a recurrent occurrence of seizures stemming from an abnormal, excessive, and/or synchronous neuronal activity in the brain due to an imbalance between excitation and inhibition of cortical areas [1,2]. While in most cases, antiseizure medications (ASM) can completely control epilepsy and render patients seizure-free, approximately 30% of patients will develop Drug-Resistant Epilepsy (DRE) [3]. Clinicians can propose resection of the epileptogenic focus for these patients under the condition that the epilepsy (i) does not present a multi-focal seizure onset zone, (ii) is not generalized, and (iii) does not have a seizure onset zone that lies in the eloquent cortex. When one of these conditions is not fulfilled, clinicians can consider neuromodulation as an adjunctive treatment to ASM, and Vagus Nerve Stimulation (VNS) is among the available options. VNS consists of the implantation of an electrode around the left vagus nerve and a pulse generator located under the left clavicula, sending electrical pulses that depolarize the nerve fibers and trigger action potentials. A previous retrospective study that included 436 patients aged 1–76 years at the time of the implantation showed that 63.75% of patients became responders, corresponding to a >50% reduction in seizure frequency [4].

While VNS has been used for over three decades, its precise mechanisms of action and the biological prerequisites for responding to the treatment are not fully understood. The activation of a group of subcortical and cortical brain regions composing the vagal afferent network is presumed to be the key component inducing antiseizure effects. In particular, VNS can modulate the activity of the thalamus and, in turn, disrupt the genesis of pathological activity in the brain [[5], [6], [7], [8], [9], [10]].

Using resting-state functional Magnetic Resonance Imaging (MRI), increased connectivity of the thalami to the anterior cingulate cortex and the left insula, as assessed before the implantation, was associated with better seizure control with VNS in children with DRE [11]. Based on these results, a Support Vector Machine (SVM) model was built and was able to discriminate responders from patients with a <50% reduction in seizure frequency with an accuracy of 88% in an external cohort of patients [11]. The potential role of the thalamus in mediating VNS efficacy was strengthened using Diffusion Tensor Imaging (DTI) to assess the brain microstructure in DRE patients [12]. Larger Fractional Anisotropy (FA, i.e., a marker of structural integrity) was found in different tracts in responders to the therapy compared to non-responders, including within left thalamocortical, limbic, and association fibers. Using DTI metrics in these tracts, an SVM correctly classified patients based on their responses with a classification accuracy of 89.5%.

Here, we argue that the role of the thalamus in VNS efficacy may be better understood using diffusion MRI, a powerful imaging technique for characterizing the microstructure of white matter tracts that remains underexploited in the field of DRE. To our knowledge, while the previous study we referred to used single-shell diffusion MRI [12], no study used high-gradient multi-shell diffusion MRI to extract microstructural features of white matter tracts in DRE patients implanted with a VNS device. Multi-shell diffusion MRI uses multiple b-values (corresponding to different gradient strengths and durations) and allows the assessment of more complex aspects of the tissue microstructure using multi-compartment models, compared with classical models such as DTI. Therefore, in the present study, we aimed to assess whether microstructural features of subsegments of the thalamocortical tracts, extracted based on multi-shell diffusion, could help to distinguish patients responding and not responding to VNS. These investigations are needed to better understand interindividual differences existing among patients that could be linked to the therapeutic efficacy of VNS. Moreover, we explored whether multi-compartment models could provide better discrimination between patients compared to single-compartment models. We hypothesize that a higher integrity of thalamocortical tracts will be found in patients demonstrating greater therapeutic efficacy. Finally, this study aims at creating a SVM model to classify patients based on their response to VNS. Clinical features (demographic and epilepsy-related features) were added to the model, to evaluate the ability of clinical data to support and improve the classification of patients based on their response to VNS.

## Material and Methods

### Participants

Patients were recruited from the Center for Refractory Epilepsy of Saint-Luc University Hospital. Inclusion criteria were (i) adult participants, (ii) with a diagnosis of DRE (persistent seizures despite the use of at least two ASM administered at correct dosages) (iii) able to understand the study protocol, (iv) implanted for at least six months with one of the following VNS models: DemiPulse® Model 103, DemiPulse Duo® Model 104, AspireHC® Model 105, or AspireSR® Model 16 (LivaNova, Inc., London, UK), and (v) patients whose medication did not influence assessment of VNS response. Exclusion criteria were: occurrence of a seizure <24h prior to the MRI acquisitions, severe side effects of VNS reported by the patients such as dyspnea, pain in the neck/ear region, or gastrointestinal complaints, history of alcohol or drug abuse, the presence of psychiatric illness, inability to understand the study protocol, and any MRI contraindication. Response to VNS was determined by the reference neurologist with the following criteria: patients are considered as Responders (R) if a >50% reduction in seizure frequency is observed, Partial Responders (PR) demonstrate a reduction in seizure frequency between 30 and 50% with positive effects observed when swiping the magnet in front of the generator manually, and Non-Responders (NR) present a <30% reduction in seizure frequency. The reference neurologist estimated response to VNS based on the seizures reported over a three-month period before the implantation. Moreover, a screening of medical records was conducted to ensure that no change in medication could have positively influenced the evaluation of response to VNS. In the present study, 18 patients were recruited, including 6 R, 5 PR, and 7 NR. Since therapeutic effects were found in PR – although, to a lower extent, these patients were grouped with R for statistical analyses. Demographic data can be found in Table 1. In three NR, the VNS was off for several reasons: (i) two patients were explanted four months and 2.4 years before the experiment, and (ii) in one patient, the device was turned off completely for almost two years due to side effects and a lack of response. The study received approval by the Ethical Committee of Saint-Luc University Hospital (reference nr. 2021/18FEV/086). All patients signed the informed consent prior to any investigation.

**Table 1:** Demographic and clinical characteristics of the study population.

Characteristics	NR (n ​= ​7)	R/PR (n ​= ​11)	p-value
Age (years)	37.14 ​± ​12.82	37.09 ​± ​8.71	0.61
Sex	3 females – 4 males	7 females – 4 males	0.63
VNS therapy duration (months)	109.28 ​± ​46.98	61.27 ​± ​71.50	0.10
Epilepsy type	7 focal – 0 generalized	9 focal – 2 generalized	0.50
Epilepsy duration (years)	24.71 ​± ​8.85	26.64 ​± ​13.09	0.84
Epilepsy onset age (years)	16.71 ​± ​10.14	10.45 ​± ​8.00	0.18
Epileptic syndrome	Lennox-Gastaut: 1/7	Lennox-Gastaut: 1/11	1.00
	No syndrome: 6/7	No syndrome: 10/11	
Brain surgery	Brain surgery: 3/7	Brain surgery: 3/11	0.62
	No surgery: 4/7	No surgery: 8/11	
Etiology of epilepsy	Structural: 3/7	Structural: 4/11	0.76
	Viral: 1/7	Viral: 0/11	
	Genetic: 0/7	Genetic: 2/11	
	Unknown: 3/7	Unknown: 5/11	
Lesion on brain MRI	Lesion: 5/7	Lesion: 5/11	0.37
	No lesion: 2/7	No lesion: 6/11	
Number of ASMs	2 ASMs: 2/7	2 ASMs: 5/11	0.30
	3 ASMs: 2/7	3 ASMs: 5/11	
	4 ASMs: 3/7	4 ASMs: 1/11	
Benzodiazepine (daily) intake (number of patients)	2	1	0.53
VNS intensity (mA)^a^	1 ​mA: 0/4	1 ​mA: 1/11	0.05
	1.125 ​mA: 0/4	1.125 ​mA: 1/11	
	1.25 ​mA: 0/4	1.25 ​mA: 1/11	
	1.50 ​mA: 0/4	1.50 ​mA: 5/11	
	1.75 ​mA: 2/4	1.75 ​mA: 1/11	
	2 ​mA: 2/4	2.00 ​mA: 2/11	
VNS frequency (Hz)^a^	20 ​Hz: 3/4	20 ​Hz: 4/11	0.21
	25 ​Hz: 1/4	25 ​Hz: 2/11	
	30 ​Hz: 0/4	30 ​Hz: 5/11	
VNS pulse width (μs)^a^	250 μs : 3/4	250 μs : 9/11	1.00
	500 μs : 1/4	500 μs : 2/11	
Rapid duty cycle^a^^,^^b^	0/4	2/11	0.58

NR: Non-Responder, R: Responder, PR: Partial Responder, ASM: Antiseizure medication.

a Values reported after excluding three NR (see text).

b The duty cycle is defined as (ON time ​+ ​4s)/(ON time ​+ ​OFF time), and a rapid duty cycle is defined as an OFF time <1.1 ​min while keeping the duty cycle <50% [41].

### Imaging parameters

Imaging data were acquired following the LivaNova guidelines for MRI. Before entering the MRI room, the output current of the VNS device was set to 0 mA, and the *AutoStim* mode was turned off. Imaging data were acquired using the SIGNA Premier 3T MRI system (GE Healthcare, Milwaukee, WI, USA) with a 48-channel head coil. T1-anatomical images were acquired with a Magnetization Prepared – RApid Gradient Echo (MPRAGE) sequence: TR = 2186 ms, TE = 2.95 ms, FA = 8°, TI = 900 ms, bandwidth = 244.14 Hz, matrix size = 256 × 256, 156 axial slices, imaging frequency = 127.77 Hz, voxel size = (1 × 1 x 1)mm^3^, acquisition time = 5:26 min. Diffusion MRI data were acquired with a Pulsed Gradient Spin Echo (PGSE) sequence: TR = 4837 ms, TE = 80.5 ms, and flip angle = 90°. A high-gradient multi-shell diffusion scheme was used and consisted of 64 gradients at b = 1000 s⋅mm^−2^ and 32 gradients at b = 2000, 3000, and 5000 s⋅mm^−2^, interleaved with 7 b0 images. The in-plane field-of-view was (220 × 220)mm^2^, the matrix size was 110 × 110, and the data contained 68 axial slices with a 2-mm thickness (no inter-slice gap, 2-mm isotropic voxels). A multi-slice excitation scheme was used during the acquisition with a hyperband slice factor of three to reduce the acquisition time. The total acquisition time was 13:33 min. A T2-weighted image was acquired to improve the patient-specific segmentation of cortical areas. The T2-weighted image was acquired using a Spin-Echo (SE) sequence: TR = 2.5 ms, TE = 91 ms, FA = 90°, matrix size = 255 × 255, 141 sagittal slices, voxel size = (1 × 1 x 1)mm^3^, acquisition time = 2:01 min.

## Data Analysis

### Preprocessing and diffusion models

Preprocessing of the diffusion data was performed using the ElikoPy pipeline (https://github.com/Hyedryn/elikopy) [13]. The preprocessing steps included skull stripping (using the Diffusion Imaging in Python library – DiPy, https://dipy.org/) [14], Rician denoising (Marchenko-Pastur Principal Component Analysis [15]), Eddy currents correction, susceptibility distortion correction and motion correction [16].

DTI maps were computed using DiPy. The DTI metrics investigated in this study included Functional Anisotropy (FA), Mean Diffusivity (MD), Axial Diffusivity (AD), and Radial Diffusivity (RD). Multi-compartment diffusion models were used to characterize crossing fascicles, i.e., Neurite Orientation Dispersion and Density Imaging - NODDI and Microstructure Fingerprinting – MF, a model known to provide near-ground truth for diffusion-weighted MRI signals and compute metrics that are biologically more interpretable [17,18]. NODDI maps were computed with the Diffusion Microstructure Imaging in Python (DMIPY, https://github.com/AthenaEPI/dmipy) toolbox [19]. Two NODDI metrics were investigated: the Intracellular Volume Fraction (ICVF), also known as the Neurite Density Index (NDI), and the Orientation Dispersion Index (ODI). This model requires two values that are fixed a priori: the isotropic diffusivity for the cerebrospinal fluid (CSF) (default value of 3∗10^−3^ mm^2^s^−1^) and the axial diffusivity of the intra-neurite space (default value of 1.7∗10^−3^ mm^2^ s^−1^). Finally, MF is a multi-compartment model based on Monte Carlo simulations of the random walk of water molecules within the brain. The MF metric computed in the present study is the weighted Fiber Volume Fraction (wFVF) corresponding to the axonal density of the fibers and is defined as:

$${wFVF}_{i}=\frac{\nu_{1,i}*{fvf}_{1,i}+\nu_{2,i}*{fvf}_{2,i}}{\nu_{1,i}+\nu_{2,i}}\text{,}$$

where:

- *i* is the index of the voxel.
- $\nu_{1,i\;}$ is the fraction of occupancy of fascicle 1 in the voxel i (and $\nu_{2,i\;}$ for fascicle 2).
- ${fvf}_{1,i}$ is the fiber volume fraction of fascicle 1 in the voxel i (and ${fvf}_{2,i}$ for fascicle 2).

### Tractography

A Fiber Orientation Distribution (FOD) was estimated with the Multi-Shell Multi-Tissue Constrained Spherical Deconvolution (MSMT-CSD) implemented in MRtrix3 [20]. Tractography was performed with the *tckgen* function of MRtrix3 in the diffusion space of the patients. A second-order integration over FOD - a probabilistic algorithm - was used to reconstruct the streamlines. A 5-tissue-type segmented image (including cortical gray matter, subcortical gray matter, white matter, CSF and pathological tissues) was computed using the *5ttgen* command of MRtrix3 and used for constraining anatomically the tractography and improve it using biological realistic priors. Streamlines were truncated and re-tracked during the tractography to avoid poor structural terminations. Tracking parameters included: tracking step size = 0.5 × voxel size (2-mm isotropic voxel), maximum angle = 15° for all tracts except tracts projecting to the parietal lobe where 10° was used, minimum length = 2 × voxel size, maximum length = 100 × voxel size, 1000 as the maximum number of sampling trials at each point, FOD amplitude cut-off value for terminating tracks = 0.05, and the number of selected streamlines after all selection criteria have been applied = 10000.

Twelve subsegments of thalamocortical tracts were reconstructed for each subject (6 left-lateralized and 6 right-lateralized tracts), including the anterior thalamocortical tracts, the superior thalamocortical tracts, the posterior thalamocortical tracts (projecting to the parietal cortex, or the occipital cortex), and the inferior thalamocortical tracts (projecting to the temporal cortex or the insular cortex) [21]. Candidate gray matter regions used as inclusion regions for the tractography were extracted in the structural space of the subjects with Freesurfer (Linux – centOS *version 7.2*), using the T1 and T2-weighted images for the segmentation of the whole brain. All Regions-Of-Interest (ROIs) were warped into the diffusion space of the subject after registering the skull-stripped T1-weighted image to the diffusion space, using the *bbregister* function from Freesurfer, and applying the transformation parameters to the segmented ROI using the *mri_vol2vol* function from Freesurfer. This step included a 6-parameter rigid body transformation with the *-no-resample* option to avoid losing resolution due to resampling of the structural image. For the tracking, white matter inclusion regions (defined based on anatomical knowledge of thalamocortical tracts [21]) were also used as ROI to improve the tractography. These regions were obtained after registration of the parcellation from the Johns Hopkins University – International Consortium for Brain Mapping (JHU ICBM) DTI-152 Atlas in the diffusion space of the subject with a two-step process: (i) registering the template image - ICBM152 (1 × 1 x 1)mm^3^ to the T1-weighted image (using the Advanced Normalization Tools toolkit - ANTs, Penn Imaging Computing and Science Laboratory, UPenn, USA, http://stnava.github.io/ANTs/) of the subject and applying the transformation parameters to warp the white matter labeled ROI into the structural space of the subject, and (ii) applying the transformation parameters obtained when registering the T1-weighted image into the diffusion space of the subjects.

**Fig. 1:**
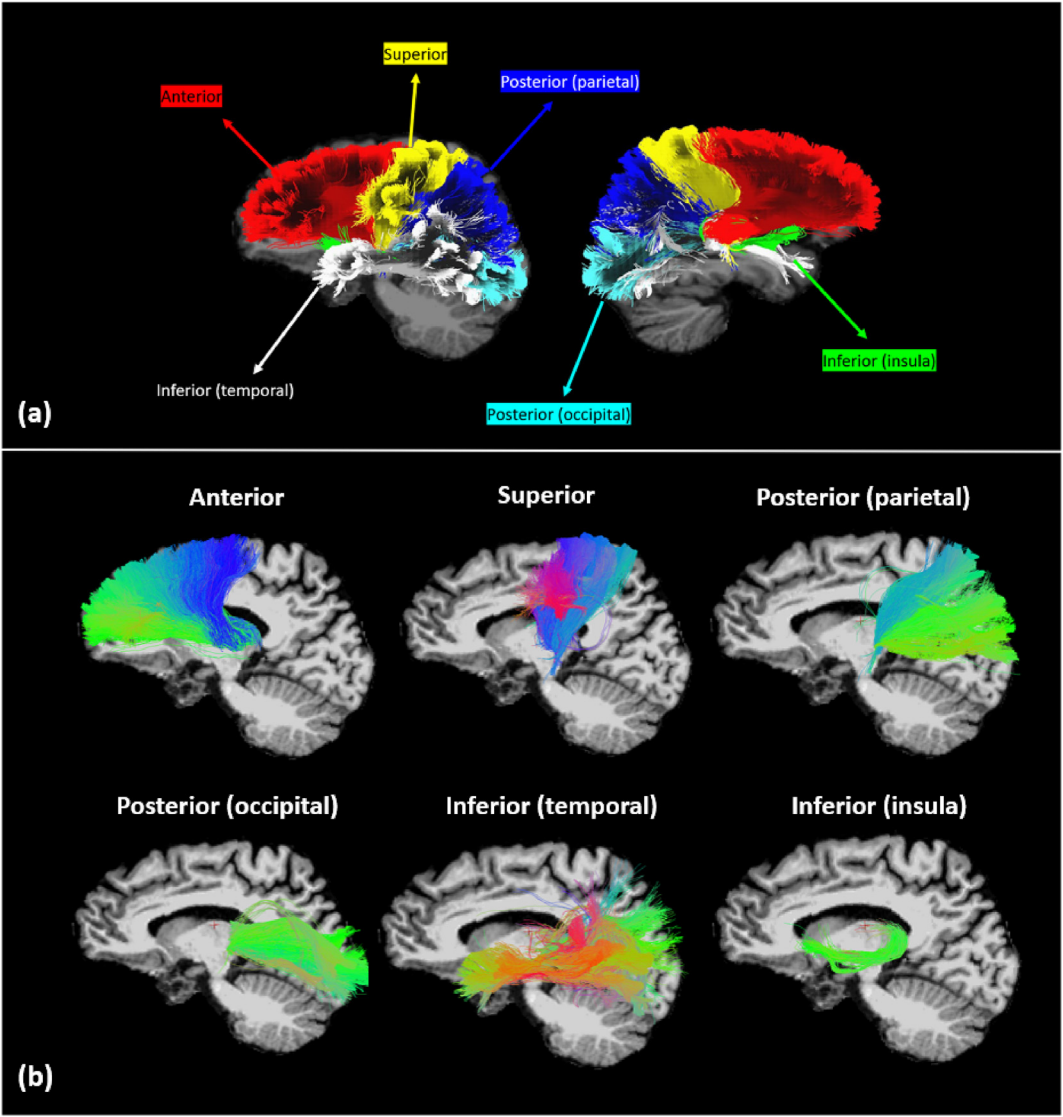
Example of the tractography of thalamocortical tracts extracted in one patient. (a) All subsegments of thalamocortical tracts superimposed with a color index, and (b) individual thalamocortical subsegments, with the color reflecting the orientation of the fibers.

For tracking all thalamocortical tracts, the Freesurfer-segmented left and right thalami were used as seed regions for tracking left and right thalamocortical tracts, respectively. The inclusion ROIs and END regions used for tracking the subsegments of the thalamocortical tracts were based on regions previously defined [21] (all regions are summarized in Table 2). The final tracts are shown in Fig. 1.

**Table 2:** Inclusion brain regions (Region-Of-Interest, ROI, and END regions) used for the tracking of thalamocortical tracts.

Thalamocortical tracts	Inclusion ROIs	END
**Anterior**	Anterior limb of the internal capsule	Frontal lobe: superior frontal cortex, rostral and caudal middle frontal cortex, pars opercularis, pars triangularis, pars orbitalis, lateral and medial orbitofrontal cortex and frontal pole
**Superior**	Posterior limb of the internal capsule	Central gyrus
**Posterior**		
Projecting to parietal lobe	Posterior limb of the internal capsule	Parietal lobe: superior parietal cortex, inferior parietal cortex and precuneus
Projecting to the occipital lobe	Posterior limb of the internal capsule	Occipital lobe: lateral occipital cortex, lingual gyrus, cuneus and pericalcarine gyrus
**Inferior**		
Projecting to the temporal lobe	Retrolenticular part of the internal capsule	Temporal lobe: superior, middle, and inferior temporal cortex, banks of the superior temporal sulcus, fusiform gyrus, transverse temporal cortex, and entorhinal cortex
Projecting to the insular cortex	Retrolenticular part of the internal capsule	Insular cortex

### Statistical analysis

Statistical analyses were conducted using RStudio (*version 4.2.1*). Demographic and clinical features summarized in Table 1 were statistically compared between R/PR and NR using Wilcoxon Mann-Whitney tests for continuous variables and Fisher's exact test for nominal data.

Using multivariate linear regressions, diffusion MRI metrics were modeled in terms of VNS response, using age, sex, ASM intake, epilepsy duration, VNS therapy duration, and benzodiazepine intake as covariates.

The Variance Inflation Factor (VIF) values were computed for the predictors included in the linear models to avoid fitting problems. VIF values of all predictors were <5 (low correlation between predictors), with a maximum VIF value of 2.06 found for the sex of the patients. False Discovery Rate (FDR) correction was applied for all the tracts investigated to correct for multiple comparisons (12 tests in total). Results were considered as significant for p_FDR_ < 0.05. Trends toward significance were considered for p < 0.05. For visual purposes, boxplots of diffusion metrics in R/PR and NR were shown. Tracking of the right posterior thalamocortical tract projecting to the parietal lobe failed for one patient (an NR) due to a right amygdalohippocampectomy associated with a resection of temporo-occipital dysplasia. Therefore, the diffusion metrics were investigated in 18 patients for all tracts, except for the right posterior thalamocortical tracts projecting to the parietal lobe, for which 17 patients were included in the analysis.

### Support vector machine

Seventeen subjects were included in the discovery cohort - the patient for whom the tractography failed for the right posterior thalamocortical tract projecting to the parietal lobe was excluded for consistency purposes in the features used to train the SVM model. An SVM model was chosen over other existing classification models, as it is known to perform well on small datasets [22]. The Scikit-Learn Python library (French Institute for Research in Computer Science and Automation, Rocquencourt, France) was used to build the SVM model [23]. Data was mean-centered and scaled to unit variance to reduce sensitivity to the feature scale and have a faster convergence when fitting the SVM model.

A recursive feature elimination approach was used to determine the optimal combination of features by removing the least important feature before refitting the model. A wrapper feature selection approach was conducted, with an internal filter-based feature selection using the *SelectKBest* function that uses F-statistics to classify features based on their contribution to the target variable – i.e., response to VNS therapy. Therefore, a model was built using all features and recursively eliminating the least important feature; the model was rebuilt until one feature (i.e., the most important feature for discriminating R/PR and NR) remained. Eighty-four diffusion MRI features were considered (12 tracts – 6 in each hemisphere; 7 metrics in total – 4 DTI metrics, 2 NODDI metrics, and 1 MF metric). Clinical features were incorporated into the set of features used for the classification to evaluate their potential influence in the classification of the patients. The following clinical features were included: age, sex, duration of VNS therapy, number of ASM, benzodiazepine intake, epilepsy duration, epilepsy onset age, epilepsy type (focal or generalized), presence of an epileptic syndrome (no syndrome or Lennox-Gastaut syndrome), etiology of epilepsy (structural, genetic, viral, or unknown), history of brain surgery, and presence of a brain lesion detected on structural MRI images. Therefore, 96 features were considered in total for building the SVM model. All clinical features that could be retrospectively extracted from medical records were added in the present study. The inclusion of these features in the model to evaluate their potential to discriminate R/PR and NR constitutes interesting investigations, as (i) response rate to VNS is known to increase with the duration of the therapy [24], (ii) white matter integrity of tracts composing the vagal afferent network may present specific abnormalities in different epilepsy types, or epilepsies with different etiologies, (iii) patients with a shorter history of epilepsy could show a higher likelihood to respond to VNS [24], and that (iv) lesions detected on MRI or history of brain surgery could explain changes in white matter microstructure in fibers composing the vagal afferent network [25,26].

A common practice in machine learning is to use kernel functions to implicitly map the data into a higher-dimensional space to solve a non-linear classification problem using a linear classifier. Therefore, different kernel functions were used during the model selection process to investigate the most suitable function for the classification: linear, polynomial, Radial Basis Functions – RBF, and sigmoid kernel functions. Grid search over the hyperparameters was realized for the regularization parameter (to ensure a trade-off between misclassifications and maximization of the margin hyperplane) and the gamma-kernel coefficient (defining the curvature of the decision boundary - only applicable for polynomial, RBF, and sigmoid kernel functions). Tuning of the hyperparameters was done with a nested Leave-One-Out (LOO) cross-validation to select the best-performing model while avoiding overfitting and bias for the estimation of the ability of the model to generalize to unseen data. Grid search over these parameters (inner cross-validation loop) was realized over classically reported values: C = [10^−4^, 10^−3^, …, 10^2^] and γ = [‘*scale’*, ‘*auto’*, 10^−4^, 10^−3^, …, 10^2^]; with *scale* = 1/(number of features ∗ data variance) and *auto* = 1/(number of features).

Due to the small nature of our dataset, the classification accuracy was also evaluated using the LOO cross-validation technique (outer cross-validation loop). The confusion matrix reporting the true positive (TP), true negative (TN), false positive (FP), and false negative (FN) predictions was computed. The final model (best subset of features, most suitable kernel function, and hyperparameters) was chosen based on a multi-criterion evaluation that includes:

- F1-score, defined as: 2∗(*precision* ∗ *recall*)/(*precision* + *recall*), with *precision* = TP/(TP + FP) and *recall* = *sensitivity* = TP/(TP + FN).
- Classification accuracy defined as: (TP + TN)/(TP + TN + FP + FN).

For the best-performing model, the Receiver Operating Characteristic (ROC) curve was plotted, and the Area-Under the Curve (AUC) was computed based on the prediction scores using the *roc_auc_score* function.

## Results

### Diffusion tensor imaging

**Fig. 2:**
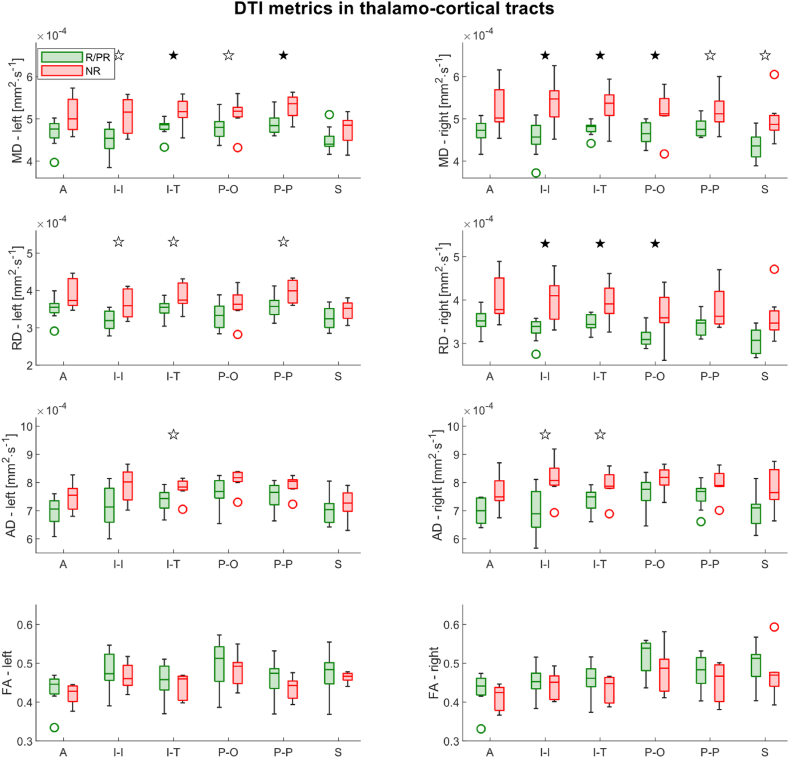
Boxplots of DTI metrics in thalamocortical tracts in Responders (R)/Partial Responders (PR) and Non-Responders (NR). Filled stars represent the results of the linear models that remained significant after FDR correction; Empty stars represent the results of the linear models that were significant without correction only. AD: Axial Diffusivity, FA: Fractional Anisotropy, MD: Mean Diffusivity, RD: Radial Diffusivity, A: Anterior thalamocortical tracts, I–I: Inferior thalamocortical tracts projecting to the insular cortex, I–T: Inferior thalamocortical tracts projecting to the temporal lobe, P–O: Posterior thalamocortical tracts projecting to the occipital lobe, P–P: Posterior thalamocortical tracts projecting to the parietal lobe, S: Superior thalamocortical tracts.

The linear model using the DTI metrics as dependent variables and controlling for several potential confounds (see methods) revealed a significantly higher MD in NR compared to R/PR bilaterally in the inferior thalamocortical tracts projecting to the temporal lobe (left: p = 0.001, p_FDR_ = 0.01∗, right: p = 0.002, p_FDR_ = 0.01∗), left posterior thalamocortical tracts projecting to the parietal lobe (p = 0.01, p_FDR_ = 0.03∗), right inferior thalamocortical tracts projecting to the insular cortex (p = 0.007, p_FDR_ = 0.02∗), and right posterior thalamocortical tracts projecting to the occipital cortex (p = 0.004, p_FDR_ = 0.01∗) (Fig. 2). Moreover, a significantly higher RD was found in NR compared to R/PR in the right inferior thalamocortical tracts projecting to the insular cortex (p = 0.007, p_FDR_ = 0.02∗), right inferior thalamocortical tracts projecting to the temporal lobe (p = 0.007, p_FDR_ = 0.02∗), and right posterior thalamocortical tracts projecting to the occipital lobe (p = 0.002, p_FDR_ = 0.02∗). Linear models of diffusion metrics of models showing significant results after FDR correction (p_FDR_ < 0.05) or trends of significance (p < 0.05) are reported in Supplementary Material 1.

Moreover, while the duration of VNS therapy was used as a covariate in the statistical models to remove a possible influence on the diffusion metrics, no significant effect of therapy duration was found in any statistical model.

### Neurite orientation dispersion and density imaging

**Fig. 3:**
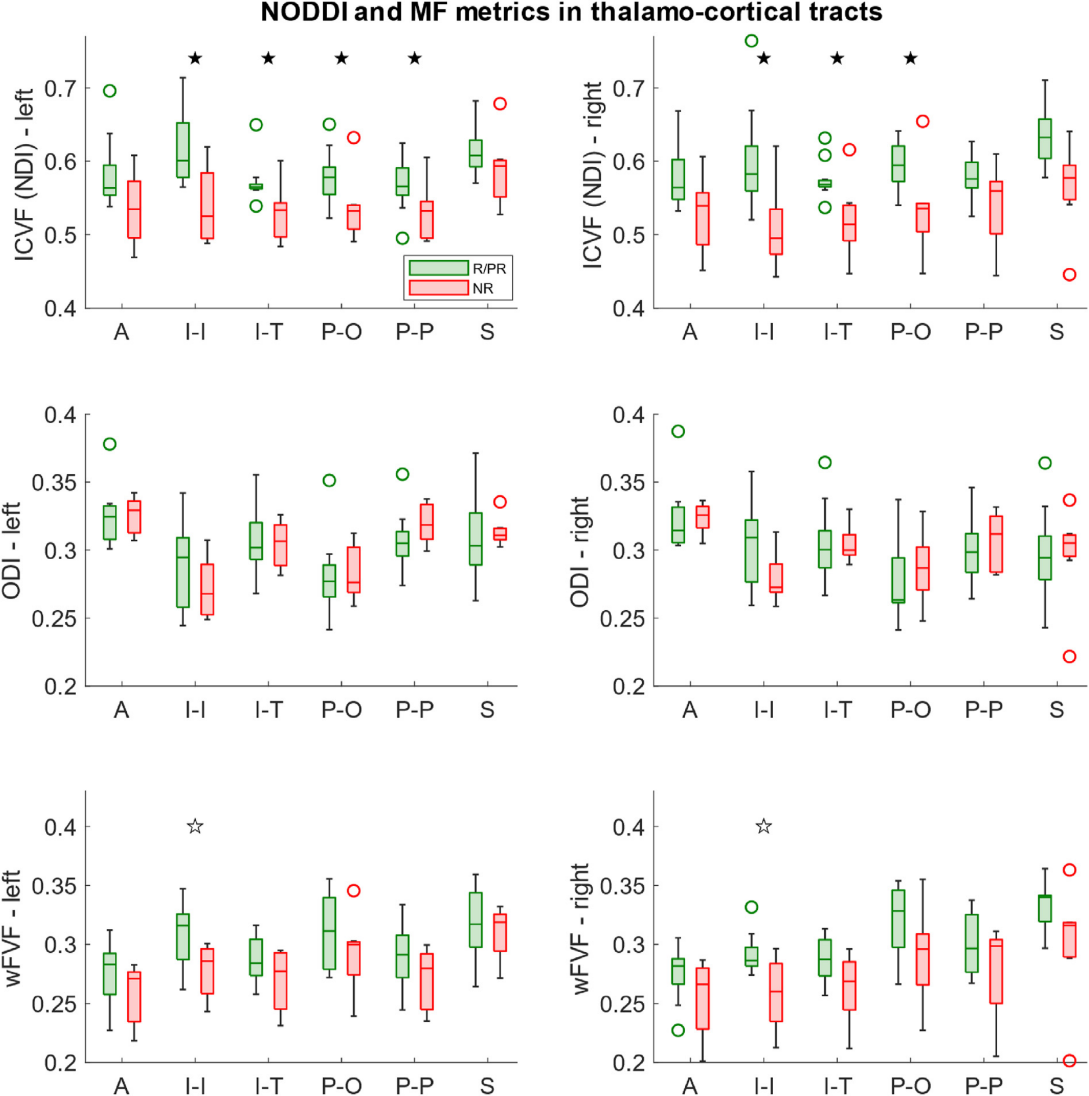
Boxplots of NODDI and MF metrics in thalamocortical tracts in Responders (R)/Partial Responders (PR) and Non-Responders (NR). Filled stars represent the results of the linear models that remained significant after FDR correction; Empty stars represent the results of the linear models that were significant without correction only. ICVF: Intracellular Volume Fraction, NDI: Neurite Density Index, ODI: Orientation Dispersion Index, wFVF: weighted Fiber Volume Fraction. A: Anterior thalamocortical tracts, I–I: Inferior thalamocortical tracts projecting to the insula, I–T: Inferior thalamocortical tracts projecting to the temporal lobe, P–O: Posterior thalamocortical tracts projecting to the occipital lobe, P–P: Posterior thalamocortical tracts projecting to the parietal lobe, S: Superior thalamocortical tracts.

The linear model using the NODDI metrics as dependent variables yielded a significantly higher ICVF (or NDI) bilaterally in R/PR compared to NR in the inferior thalamocortical tracts projecting to the insular cortex (left: p = 0.02, p_FDR_ = 0.04∗, right: p = 0.01, p_FDR_ = 0.04∗), inferior thalamocortical tracts projecting to the temporal lobe (left: p = 0.005, p_FDR_ = 0.02∗, right: p = 0.004, p_FDR_ = 0.02∗), posterior thalamocortical tracts projecting to the occipital lobe (left: p = 0.02, p_FDR_ = 0.04∗, right: p = 0.004, p_FDR_ = 0.02∗), and left posterior thalamocortical tracts projecting to the parietal lobe (p = 0.02, p_FDR_ = 0.04∗) (Fig. 3).

### Microstructure fingerprinting

The linear model for the MF metrics led to no significant difference after FDR correction. However, a trend toward a higher wFVF in R/PR compared to NR was found bilaterally in the inferior thalamocortical tracts projecting to the insular cortex (left: p = 0.04, right: p = 0.02) (Fig. 3).

### Support vector machine

The feature selection technique highlighted different models that led to the highest classification accuracy and the associated F1-score. The best model that included the least number of features only used the five best discriminatory features for the classification. This model reached a classification accuracy of 94.12% and an F1-score of 95.65%. The selected features were: MD in left inferior thalamocortical projecting to the insular cortex (score_SelectKBest_: 12.25, p_SelectKBest_ = 0.003), MD in right inferior thalamocortical tracts projecting to the insular cortex (score_SelectKBest_: 11.50, p_SelectKBest_ = 0.004), RD in right inferior thalamocortical tracts projecting to the insular cortex (score_SelectKBest_: 11.11, p_SelectKBest_ = 0.004), RD in left inferior thalamocortical tracts projecting to the insular cortex (score_SelectKBest_: 9.65, p_SelectKBest_ = 0.007) and MD in the right superior thalamocortical tracts (score_SelectKBest_: 9.56, p_SelectKBest_ = 0.007).

**Fig. 4:**
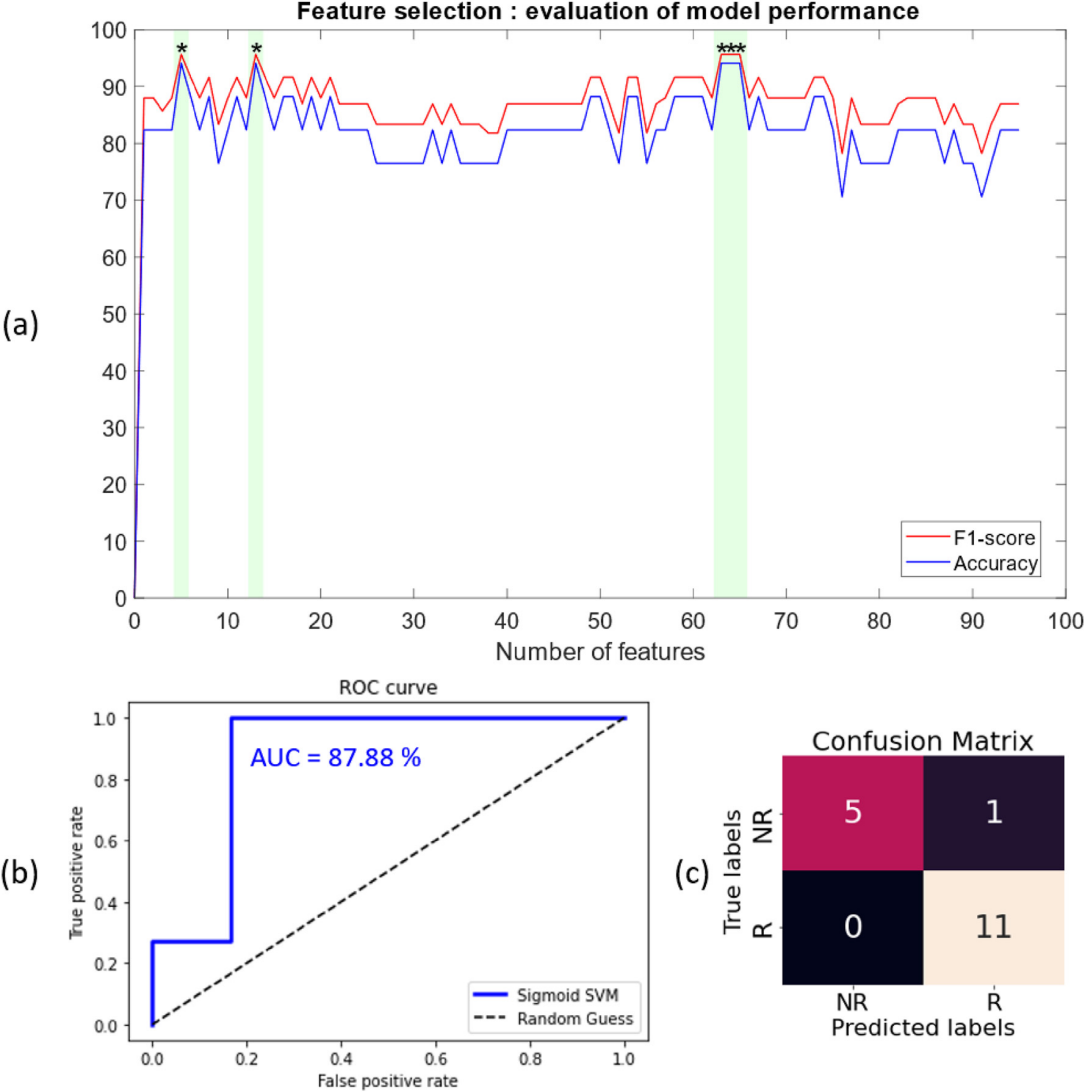
Model and feature selection techniques for building the final SVM model. (a) Evolution of the classification accuracy and F1-score of the best-performing model during the recursive feature elimination; (b) Receiver Operating Characteristics (ROC) curve of the final Support Vector Machine (SVM) classifier, with an Area Under the Curve (AUC) of 87.88%; (c) Confusion matrix of the final SVM model, showing 5 true negative (TN) predictions, 11 true positive (TP) predictions, 0 false negative (FN) prediction and one false positive (FP) prediction.

The same performance (classification accuracy and F1-score) was found when using the 13, 63, 64, and 65 best features (Fig. 4a). Moreover, the accuracy remained high regardless of the number of features chosen for the classification, providing evidence of stability of the model, and hints that the model is not overfitting. The classification accuracy and F1-score remained consistently high during the features selection technique, indicating the stability of the selected model when features are added for the classification. Hence, to (i) prevent overfitting, (ii) optimize the computational efficiency, and (iii) enhance the generalization properties of the model, the final model chosen was the one showing the best performance and the minimal number of features for the classification (i.e., 5 best discriminatory features). At each iteration of the LOO cross-validation technique, the sigmoid kernel was selected as the best-performing model, and grid-search over the hyperparameters suggested a top-performing classification for the default values of C = 1, and γ = 1/(number of features ∗ data variance) = 0.2. These values promote good generalization by balancing the complexity and smoothness of the decision boundary, which is essential for preventing overfitting in small datasets. Using this model, the AUC = 87.88%, the sensitivity = 100%, and the specificity = 83.33%. The corresponding ROC curve and confusion matrix can be found in Fig. 4b and c, respectively.

The most discriminatory clinical feature selected was the number of ASM, selected at the 39th position out of 96 features (with a higher number of ASM in patients with a poorer response to VNS, which was not significant when comparing groups in the demographic data table – Table 1).

## Discussion

Considering the broadness of projections arising from the thalamus and projecting to the cortex, modulation of this circuitry with VNS could disrupt the abnormal and synchronous activity of neurons [10]. The present pilot study aims to increase our current understanding of the possible implication of thalamocortical tract integrity in the interindividual differences in response to VNS using diffusion MRI. Based on the diffusion metrics extracted, our results suggested a lower integrity in different subsegments of thalamocortical tracts in patients with a poorer response to VNS.

The thalamus may be a central player in the antiseizure effects of VNS [[5], [6], [7], [8], [9]]. In addition to the rationale provided in the introduction, an immunochemistry study carried out in rodents found an increased nuclear Fos immunolabelling - a marker for high neuronal activation - in the habenular nuclei of the thalamus with VNS [5]. Moreover, previous positron emission tomography studies conducted in humans found a bilateral increased cerebral blood flow in the thalami following VNS administration [[6], [7], [8]]. Interestingly, the increased cerebral blood flow measured in one of these studies correlated with decreased seizure frequency [7]. Using functional MRI, an increased activation in different cortical areas was found with VNS, while an increase in the thalamus was reported in 2 out of 5 patients – those who demonstrated improvement in seizure control after the implantation [9]. Moreover, previous studies explored the connectivity features of thalamocortical relays in DRE patients [5,[7], [8], [9],^11^]. Indeed, a functional MRI study found an increased connectivity of the thalami to the anterior cingulate cortex and the left insula before the implantation, which was associated with a higher therapeutic efficacy of VNS [11]. Finally, a previous diffusion MRI study found an increased FA in the left thalamocortical, limbic, and association fibers in responders to the therapy compared to patients with a <50% reduction in seizure frequency [12].

In the present study, higher MD and RD were found mainly in subsegments of the inferior and posterior thalamocortical tracts in NR. Although not specific, higher MD and RD could indicate reduced fiber integrity, including a reduced density of axons and/or lower myelination [[27], [28], [29]]. Since DTI may suffer from strong assumptions and poor specificity, multi-compartment models were used to give further insights into microstructural differences across DRE patients.

High-gradient multi-shell diffusion MRI allowed us to use NODDI and MF, two multi-compartment models, for extracting microstructural metrics. While NODDI has been used to characterize white matter microstructure in an array of neurologic and psychiatric diseases, including Alzheimer's disease [30], Parkinson's disease [31], epilepsy [25], or schizophrenia [32], this study constitutes the first investigations of NODDI and MF metrics in DRE patients implanted with a VNS device. The lower NODDI-based ICVF found in subsegments of the inferior and posterior thalamocortical tracts in NR could suggest that the lower integrity suspected based on MD and RD may arise from a reduced fiber density. Since trends toward lower MF-based wFVF values were also found in the inferior thalamocortical tracts (projecting to the insular cortex), the results from the MF analysis further support the interpretation of the results obtained from the other diffusion models. Therefore, this pilot study could suggest that metrics from multi-compartment models may provide additional insight into microstructural differences without suffering from the strong assumptions of DTI.

The accuracy of 94.12% reached with the best SVM model only included classical DTI metrics, suggesting that they may be the best diffusion metrics to date to support clinical decisions. The best features selected for the SVM classification mainly involved diffusion metrics in the inferior thalamocortical tracts projecting to the insular cortex, i.e., tracts that also showed significant differences between R/PR and NR in the multiple regression analyses. Interestingly, a functional MRI study of the acute effects of VNS found a bilateral activation of the thalami – but more robustly left-lateralized – and insular cortices, suggesting the involvement of these brain areas in the antiseizure effects of VNS [33]. Since a lower integrity of white matter tracts connecting these regions was found in non-responders in the present study, one could suggest that a lower integrity of these tracts could result in a lower therapeutic efficacy due to possibly impaired communication. While only DTI metrics were selected for the SVM model, the statistical models that included multi-compartment features provided additional information on the microstructural properties of thalamocortical tracts and confirmed the interpretation of DTI metrics. However, more research is necessary to determine whether multi-shell diffusion MRI could guide clinical decisions in the future.

Importantly, the best SVM model did not select clinical features. These findings reinforce the idea that clinical characteristics may not be useful for improving the prediction of VNS response [12]. In line with our results, another 11-year retrospective study that included 365 pediatric patients built a prediction model and suggested that clinical features alone were not sufficient to accurately predict VNS response [34]. Furthermore, this reflects the value of diffusion MRI metrics as markers of VNS response and the potential use of our classification model to patients implanted with a VNS device for whom clinical data is incomplete. Indeed, the performance of this model will remain unaffected by challenges related to the availability of clinical data. Our findings are similar to a previous DTI study where an SVM model was built (using metrics from various white matter tracts based on a tract-based spatial statistics analysis) and reached a classification accuracy of 89.5% when discriminating NR and R. Adding clinical features did not improve the classification [12]. This contrasts with another study that used EEG-based connectivity measures to build a classification model and suggested that clinical data were useful for predicting VNS response [35]. Although the comparison between techniques and studies is difficult, one could argue that DTI connectivity metrics are more powerful than EEG connectivity metrics in predicting response to VNS, as they may not require a full clinical characterization of the patients. Future prospective studies including an extensive clinical characterization of the patients could confirm these findings. Indeed, a previous study indicated that IQ deficiency may be a predictor for responding to VNS [36]. However, including this feature – among others – in the classifier developed by Mithani and colleagues, did not improve the classification of patients [12].

Although the number of patients included in the present study is considered acceptable for a population of DRE patients implanted with a VNS device, it is important to note that the sample size remains relatively small. Therefore, while the present pilot study aimed at investigating differences of structural integrity in a pathway involved in antiseizure effects of VNS between patients showing a good and poor response to the treatment, a replication of these results is highly needed in a more extensive cohort of patients with DRE. Moreover, using an external cohort of patients to validate the SVM classifier built in the present study is warranted to evaluate the generalization, robustness, and practical relevance of the model. Besides these limitations, which are intrinsic to our study design, whether the differences observed in the present study (i) reflect an inherent inclination to respond more favorably to VNS, (ii) are linked to seizure activity - given that patients with a poorer response to the treatment may tend to experience seizures more frequently - or (iii) reflect the direct and more prominent impact of VNS in reorganizing tracts in patients with a better response, remains to be clarified. Indeed, a previous study suggested an increased MD in patients with drug-resistant temporal lobe epilepsy compared to controls in fasciculi carrying temporal lobe connections that could reflect astrogliosis and microstructure derangement related to seizure activity in the vicinity of the seizure focus [37,38]. One of these studies found a higher MD that was associated with a shorter interval between the last seizure and DTI [38]. In their study, 24 patients had an average of 50 ± 54h between the last seizure and DTI examination, and 6 patients underwent MRI acquisition as outpatients: 5 had a seizure one week before the examination, and one patient had a seizure 10 min before [38]. While our exclusion criteria involved excluding patients who had a seizure within 24h prior to the MRI examination to control for the possible impact of seizures on microstructural derangement, further studies incorporating hospitalized patients could provide insight into the potential influence of seizures on diffusion metrics of multi-compartment models. For our study design, knowing the exact time of the latest seizure prior to the MRI examination could be useful to control for possible seizure-related effects. Moreover, including a benchmark cohort of epileptic patients characterized by the same seizure burden as our cohort could be valuable for future research to extract the effect of VNS and remove possible effects of seizures on thalamocortical integrity. Further studies could only include patients thoroughly maintaining a seizure diary to precisely evaluate the reduction of seizure frequency. This could help to refine the categorization of patients, by using a continuous variable characterizing VNS response instead of using a binary classification. Longitudinal studies assessing diffusion metrics both before the implantation and throughout the treatment could be interesting to evaluate the modulatory effect of VNS on the integrity of thalamocortical tracts. Interestingly, the effect of different stimulation paradigms on thalamocortical integrity could be explored longitudinally. These investigations could help to better select stimulation parameters to maximize the neuromodulatory effects of VNS. For example, microburst stimulation – a paradigm that aims to stimulate with high-frequency bursts of stimulation – is believed to improve the modulation of the thalamus [39,40]. Comparing the effect of microburst VNS on thalamocortical integrity with classical stimulation paradigms could validate the potential of microburst VNS to improve neuroplasticity effects in the brain. However, further studies are needed to evaluate the potential effect of this paradigm in improving the response to VNS.

Pre-implantation acquisitions are needed to evaluate the value of diffusion metrics within thalamocortical tracts to predict VNS response. However, this pilot study constitutes interesting insights into the variability in response across implanted patients. Finally, investigations of the structural-functional associations in DRE patients using a multimodal approach that includes high-gradient multi-shell diffusion MRI and, for instance, functional MRI could refine the understanding of the biological prerequisites for responding to VNS.

Overall, our study highlighted the significant potential of single- and multi-compartment diffusion MRI models in elucidating interindividual differences in biological features that could be associated with VNS response. Investigation of the predictive value of multi-compartment models in a clinical context, and their potential to unravel the neuromodulatory effects of VNS on thalamocortical tracts could be investigated in pre-implantation and longitudinal studies utilizing the methodology described in this pilot study. These imaging techniques could contribute to medical decision-making, patient management, and the innovation of novel clinical treatments in the future.

## Author contributions

AB, GV, LD and RET conceived and designed the study. AB was involved in data collection, methodology design, data analysis and drafting of the original manuscript. ND was involved in methodology design and data analysis. MC was involved in data analysis. All authors were involved in the interpretation of the data and the review of the manuscript.

## Declaration of competing interest

The authors declare the following financial interests/personal relationships which may be considered as potential competing interests: Alexandre Berger was an employee of Synergia Medical SA. The firm was not involved in the study design, data collection and analysis, interpretation of the data, the writing of the article, or the decision to submit it for publication. The remaining authors declare that the research was conducted in the absence of any commercial or financial relationships that could be construed as a potential conflict of interest.
