# Supplementary material for: Identifying responders to vagus nerve stimulation based on microstructural features of thalamocortical tracts in drug-resistant epilepsy
Source: Neurotherapeutics. 2024 Jul 4;21(5):e00422. doi: 10.1016/j.neurot.2024.e00422 (PMC11579871; doi:10.1016/j.neurot.2024.e00422)
Supplement: Multimedia component 1 [file mmc1.docx]

**Supplementary Material 1**: Linear models investigating the relationship between diffusion metrics and VNS response, correcting for age, sex, ASM intake, benzodiazepine intake, epilepsy duration, and VNS therapy duration. Models showing a significant relationship (p_FDR_ < 0.05) or a trend toward significance (p < 0.05) between diffusion metrics and VNS response are shown here (uncorrected p-values reported). Refer to text for FDR-corrected p-values of significant results.

| **Diffusion metric ~** | **Response** | **Age** | **Sex** | **ASM** | **Benzo** | **Epilepsy duration** | **Duration VNS therapy** |
| --- | --- | --- | --- | --- | --- | --- | --- |
| **DTI** |  |  |  |  |  |  |  |
| ☆ MD Left I-I | p = 0.03  t = -2.43 | p = 0.17  t = -1.48 | p = 0.99  t = 0.002 | p = 0.54  t = -0.63 | p = 0.98  t = -0.02 | p = 0.78  t = 0.28 | p = 0.44  t = 0.80 |
| ★ MD Left I-T | p = 0.001  t = -4.44 | p = 0.01  t = -3.05 | p = 0.10  t = -1.82 | p = 0.11  t = -1.72 | p = 0.11  t = -1.76 | p = 0.63  t = 0.49 | p = 0.30  t = 1.10 |
| ☆ MD Left P-O | p = 0.03  t = -2.48 | p = 0.04  t = -2.44 | p = 0.08  t = -1.97 | p = 0.11  t = -1.76 | p = 0.27  t = -1.16 | p = 0.09  t = 1.90 | p = 0.10  t = 1.82 |
| ★ MD Left P-P | p = 0.01  t = -2.95 | p = 0.06  t = -2.16 | p = 0.22  t = -1.30 | p = 0.14  t = -1.59 | p = 0.64  t = -0.48 | p = 0.19  t = 1.42 | p = 0.45  t = 0.79 |
| ★ MD Right I-I | p = 0.007  t = -3.35 | p = 0.20  t = -1.37 | p = 0.96  t = -0.05 | p = 0.47  t = -0.75 | p = 0.47  t = -0.74 | p = 0.83  t = -0.21 | p = 0.69  t = 0.41 |
| ★ MD Right I-T | p = 0.002  t = -4.20 | p = 0.04  t = -2.36 | p = 0.33  t = -1.01 | p = 0.13  t = -1.66 | p = 0.23  t = -1.26 | p = 0.51  t = 0.67 | p = 0.77  t = 0.30 |
| ★ MD Right P-O | p = 0.004  t = -3.74 | p = 0.04  t = -2.40 | p = 0.08  t = -1.99 | p = 0.08  t = -1.95 | p = 0.10  t = -1.80 | p = 0.24  t = 1.24 | p = 0.23  t = 1.28 |
| ☆ MD Right P-P | p = 0.03  t = -2.58 | p = 0.04  t = -2.35 | p = 0.35  t = -0.98 | p = 0.49  t = -0.72 | p = 0.27  t = -1.17 | p = 0.27  t = 1.18 | p = 0.62  t = 0.51 |
| ☆ MD Right S | p = 0.04  t = -2.28 | p = 0.39  t = -0.90 | p = 0.75  t = -0.32 | p = 0.59  t = 0.56 | p = 0.30  t = -1.10 | p = 0.98  t = 0.03 | p = 0.32  t = 1.04 |
| ☆ RD Left I-I | p = 0.03  t = -2.49 | p = 0.11  t = -1.78 | p = 0.96  t = -0.04 | p = 0.42  t = -0.84 | p = 0.92  t = 0.10 | p = 0.47  t = 0.75 | p = 0.52  t = 0.67 |
| ☆ RD Left I-T | p = 0.02  t = -2.65 | p = 0.14  t = -1.60 | p = 0.49  t = -0.72 | p = 0.43  t = -0.82 | p = 0.19  t = -1.41 | p = 0.90  t = 0.12 | p = 0.85  t = 0.20 |
| ☆ RD Left P-P | p = 0.03  t = -2.43 | p = 0.17  t = -1.47 | p = 0.69  t = -0.41 | p = 0.29  t = -1.12 | p = 0.73  t = -0.35 | p = 0.35  t = 0.97 | p = 0.80  t = 0.26 |
| ★ RD Right I-I | p = 0.007  t = -3.38 | p = 0.17  t = -1.47 | p = 0.93  t = 0.09 | p = 0.46  t = -0.76 | p = 0.52  t = -0.66 | p = 0.89  t = -0.14 | p = 0.73  t = 0.35 |
| ★ RD Right I-T | p = 0.007  t = -3.39 | p = 0.13  t = -1.64 | p = 0.73  t = -0.34 | p = 0.35  t = -0.98 | p = 0.20  t = -1.37 | p = 0.61  t = 0.52 | p = 0.94  t = 0.07 |
| ★ RD Right P-O | p = 0.002  t = -4.04 | p = 0.04  t = -2.39 | p = 0.31  t = -1.06 | p = 0.14  t = -1.60 | p = 0.03  t = -2.54 | p = 0.29  t = 1.11 | p = 0.69  t = 0.40 |
| ☆ AD Left I-T | p = 0.04  t = -2.29 | p = 0.09  t = -1.94 | p = 0.15  t = -1.54 | p = 0.24  t = -1.25 | p = 0.80  t = -0.26 | p = 0.56  t = 0.60 | p = 0.23  t = 1.28 |
| ☆ AD Right I-I | p = 0.03  t = -2.62 | p = 0.34  t = -0.99 | p = 0.85  t = -0.20 | p = 0.57  t = -0.59 | p = 0.53  t = -0.65 | p = 0.82  t = -0.23 | p = 0.71  t = 0.38 |
| ☆ AD Right I-T | p = 0.02  t = -2.85 | p = 0.07  t = -2.07 | p = 0.18  t = -1.45 | p = 0.11  t = -1.74 | p = 0.77  t = -0.29 | p = 0.63  t = 0.49 | p = 0.63  t = 0.50 |
| **NODDI** |  |  |  |  |  |  |  |
| ★ ICVF Left I-I | p = 0.02  t = 2.82 | p = 0.08  t = 1.94 | p = 0.71  t = -0.38 | p = 0.32  t = 1.05 | p = 0.98  t = 0.03 | p = 0.95  t = -0.06 | p = 0.53  t = -0.65 |
| ★ ICVF Left I-T | p = 0.005  t = 3.51 | p = 0.04  t = 2.32 | p = 0.64  t = 0.49 | p = 0.15  t = 1.54 | p = 0.20  t = 1.38 | p = 0.91  t = 0.11 | p = 0.53  t = -0.64 |
| ★ICVF Left P-O | p = 0.02  t = 2.69 | p = 0.04  t = 2.32 | p = 0.36  t = 0.95 | p = 0.07  t = 2.02 | p = 0.34  t = 1.00 | p = 0.17  t = -1.47 | p = 0.23  t = -1.28 |
| ★ ICVF Left P-P | p = 0.02  t = 2.59 | p = 0.06  t = 2.13 | p = 0.49  t = 0.71 | p = 0.08  t = 1.98 | p = 0.55  t = 0.62 | p = 0.23  t = -1.29 | p = 0.58  t = -0.58 |
| ★ ICVF Right I-I | p = 0.01  t = 2.87 | p = 0.27  t = 1.16 | p = 0.81  t = -0.24 | p = 0.37  t = 0.94 | p = 0.56  t = 0.60 | p = 0.61  t = 0.52 | p = 0.92  t = -0.10 |
| ★ ICVF Right I-T | p = 0.004  t = 3.70 | p = 0.06  t = 2.11 | p = 0.76  t = 0.31 | p = 0.14  t = 1.60 | p = 0.27  t = 1.18 | p = 0.72  t = -0.37 | p = 0.92  t = -0.10 |
| ★ ICVF Right P-O | p = 0.004  t = 3.68 | p = 0.03  t = 2.46 | p = 0.30  t = 1.10 | p = 0.11  t = 1.73 | p = 0.13  t = 1.64 | p = 0.39  t = -0.89 | p = 0.26  t = -1.20 |
| **MF** |  |  |  |  |  |  |  |
| ☆ wFVF Left I-I | p = 0.04  t = 2.29 | p = 0.14  t = 1.60 | p = 0.33  t = -1.02 | p = 0.44  t = 0.81 | p = 0.61  t = -0.53 | p = 0.79  t = -0.27 | p = 0.69  t = -0.40 |
| ☆ wFVF Right I-I | p = 0.02  t = 2.69 | p = 0.19  t = 1.39 | p = 0.70  t = -0.39 | p = 0.43  t = 0.82 | p = 0.76  t = 0.32 | p = 0.74  t = 0.34 | p = 0.90  t = -0.13 |

I-I : Inferior thalamocortical tracts projecting to the insular cortex, I-T : Inferior thalamocortical tracts projecting to the temporal lobe, P-O : Posterior thalamocortical tracts projecting to the occipital lobe, P-P : Posterior thalamocortical tracts projecting to the parietal lobe, S : Superior thalamocortical tracts, MD : Mean Diffusivity, RD : Radial Diffusivity, AD : Axial Diffusivity, ICVF : Intracellular Volume Fraction, wFVF : weighted Fiber Volume Fraction. Filled stars represent results of the linear models that remained significant after False Discovery Rate (FDR) correction for multiple comparisons; Empty stars represent results of the linear models that were significant without correction only.
